# Insight into the effect of natural aging of polystyrene microplastics on the sorption of legacy and emerging per- and polyfluorinated alkyl substances in seawater

**DOI:** 10.1016/j.heliyon.2024.e40490

**Published:** 2024-11-16

**Authors:** Badreddine Barhoumi, Marc Metian, Carlos M. Alonso-Hernández, François Oberhaensli, Nikolaos Mourgkogiannis, Hrissi K. Karapanagioti, Philippe Bersuder, Imma Tolosa

**Affiliations:** a IAEA Marine Environment Laboratories, 4a Quai Antoine 1er, 98000, Principality of Monaco, Monaco; b Department of Chemistry, University of Patras, 26504 Patras, Greece

**Keywords:** Microplastics, Polystyrene, Aging, Sorption, PFAS, Marine environment

## Abstract

Microplastics (MPs) are abundant in aquatic environments and due to their small size, surface properties, and strong hydrophobicity, they can easily sorb chemicals, thus potentially acting as pollutant carriers. To date, most studies investigating the sorption of chemicals on MPs have principally focused on virgin MPs. However, MPs in the environment undergo aging effects, which changes their physical-chemical properties and aptitude to interact with chemicals, such as per- and polyfluorinated alkyl substances (PFAS) referred to as “forever chemicals”. In this study, we compared the sorption behavior of nine PFAS, exhibiting different physical-chemical properties, on virgin and naturally aged polystyrene microplastic (PS-MPs) to explore to what extent the environmental aging affects the sorption behavior of the PS-MPs for different legacy and emerging PFAS in seawater. Differences in the morphology and surface properties of aged PS-MPs were examined by infrared spectroscopy, surface area analysis, scanning electron microscopy, and X-ray diffraction. Results revealed that compared to virgin PS-MPs, aged PS-MPs exhibited morphological changes (e.g. cavities, pits, and rough surfaces) with biofilm development and signs of oxidation on the MPs surface. PFAS sorption on PS-MPs was enhanced for the aged PS-MPs compared to virgin PS-MPs with K_d_ values ranging from 327 L kg^−1^ for PFOA to 3247 L kg^−1^ for PFOS in aged PS-MPs. The difference in sorption capacity was mainly attributed to the physical-chemical changes and the adhered biofilm observed in aged PS-MPs. Results also showed that virgin PS-MPs adsorb PFAS mainly through steric hindrance, while the aged PS-MPs may involve more complex sorption mechanisms. This research provides additional insights into the ability of aged MPs as potential carriers of legacy and emerging contaminants in the marine environment.

## Highlights

• Sorption of per- and polyfluorinated compounds on natural-aged PS-MPs was first studied.
• Sorption capacity of PFAS on natural-aged PS-MPs was higher than virgin ones.
• Natural-aged and virgin PS-MPs had different sorption mechanism for PFAS.
• Aged PS-MPs act as carriers of PFAS in seawater.

## Graphical abstract

**Figure:**
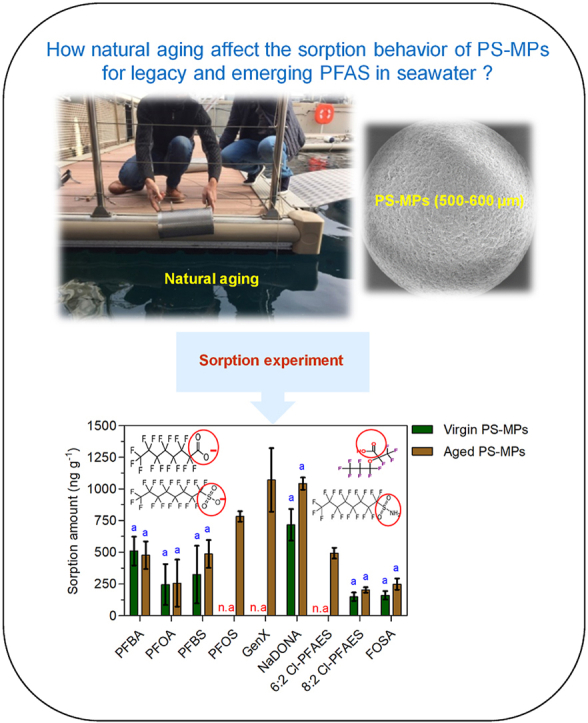

## 1 Introduction

Small-sized (<5 mm in diameter) plastics, usually defined as microplastics (MPs) occur in aquatic environments worldwide and has been increasingly recognized as a global concern [1,2]. It was estimated that about 4.85 × 10^12^ pieces of MPs accounting 35,540 tonnes were floating on the world's oceans in 2014 [3], whereas 12 billion metric tons of plastic production is presumed to be reached by 2050 [4]. Due to their small size, MPs can directly be unsafe to marine wildlife if mistakenly ingested [3,[5], [6], [7]]. Besides the adverse effects of MPs themselves, the large surface to mass ratio, strong hydrophobicity and multi-space structure of MPs make them susceptible to interact with various types of environmental chemicals [8,9], thus acting as sorbents that can alter the fate and hazard of these co-existing chemicals. In particular, other risks to organisms might occur, as chemicals sorbed to MPs may leach and bio-accumulate in different tissues of organisms when ingested [10,11]. Thus, the sorption of toxic chemicals to MPs is causing great concern.

In the aquatic environment, MPs are subject to various aging and weathering processes with physical (e.g., abrasion, degradation), chemical (e.g., solar radiation, thermal degradation, and oxidation), and biological effects (e.g., biofilm formation). These processes modify the physical-chemical properties of MPs (e.g., surface charge, roughness, hydrophobicity, polarity, and crystallinity), thus, affecting their ability to sorb chemicals [[12], [13], [14], [15], [16]]. Biological aging, which involves the formation of biofilms composed of microorganisms (e.g. bacteria, fungi, algae) and extracellular polymeric substances (EPS) on the MPs surface, was considered an important factor affecting the sorption of chemicals on MPs [15]. A recent study has showed that biofilm-covered MPs fibers exhibit a 20–85 % enhancement in the sorption of perfluorooctane sulfonate (PFOS) compared to virgin MP fibers [17]. MPs in the aquatic environment are then prone to behave differently than virgin or artificially aged MPs usually used in research laboratory experiments [18]. Laboratory accelerated aging methods (e.g., UV irradiation using lamps, hydrogen peroxide oxidation, Fenton oxidation, etc.) are widely used to investigate the sorption behavior of aged MPs [[19], [20], [21]]. Although these artificial methods revealed the critical influence of the aging process on the sorption behavior of MPs, they might be too simple to simulate much more complicated natural processes normally consisting of multiple aging factors (e.g. UV, thermal oxidation, biotic, chemical and physical effects, etc.) [22]. For example, mechanical forces such as tides and wind waves may coexist with complex mixture of organic/inorganic compounds and microbial attachment in seawater [23]. Moreover, the wavelength distribution and relative intensity of the solar light differ from those of the lamp irradiation [24]. Therefore, to further understand the environmental fate of MPs in the environment and their interaction with chemicals, new studies in laboratory conditions must consider the complex aging mechanisms that MPs can undergo in real environment to obtain more realistic conclusions.

MPs might sorb numerous legacy and emerging contaminants, comprising per- and poly-fluoroalkyl substances (PFAS) [[25], [26], [27]]. PFAS, often denoted as ‘forever chemicals’ due to their strong C–F bond (∼110 kcal/mol), have become contaminants of emerging concern because of their ubiquity, persistence, bioaccumulation potential, toxicity, and long-range transport [28]. Perfluoroalkane sulfonic acids (PFSAs) and perfluoroalkyl carboxylic acids (PFCAs) are the two major groups of PFAS detected in the environment, represented by PFOS and perfluorooctanoic acid (PFOA), respectively which are well recognized for their detrimental effects on the endocrine, reproductive, nephrotoxic, and hepatic systems [29,30]. Consequently, PFOS, PFOA and their salts were successively restricted under Annex A/B of the Stockholm Convention on Persistent Organic Pollutants (POPs) in 2009 and 2019 [31]. Meanwhile, a variety of alternatives have been introduced, including short-chain PFAS (e.g., perfluorobutanoic acid (PFBA) and perfluorobutanesulfonic acid (PFBS)), and chlorinated polyfluoroalkyl ether sulfonates (Cl-PFAES). Hexafluoropropylene oxide dimer acid (GenX), sodium dodecafluoro-3H-4,8-dioxanonanoate (NaDONA), 6:2 chlorinated polyfluoroalkyl ether sulfonates (6:2 Cl-PFAES), and 8:2 Cl-PFAES are typical examples of emerging per- and polyfluoroalkyl ether acids (PFEAs). Because of their high increase in production and usage, these new emerging PFAS have been found in different environmental compartments [[32], [33], [34]]. Thus, exploring the fate of these PFAS is essential to evaluate its impact in the marine environment.

In this study, polystyrene (PS) was chosen as a sorbent because it is one of the most widely used polymer due to its excellent performance and wide application [35,36]. Only a few studies to date have explored the interactions between PFAS and PS-MPs [[37], [38], [39], [40], [41]] and PFAS and other types of MPs [17,27,[42], [43], [44], [45], [46], [47]]. However, no studies are available in literature that investigated the sorption behavior of PFAS on naturally aged PS-MPs in seawater. In this framework, we studied the influence of natural aging on the sorption capacity of PS-MPs using some representative PFAS, with the aim to advance our knowledge on the environmental impact of these two co-existing emerging pollutants.

## 2 Materials and methods

### 2.1 Chemicals, reagents and materials

Nine PFAS with different physicochemical properties were selected as target sorbates for the sorption experiments: two PFCAs (PFBA and PFOA), two PFSAs (PFBS and PFOS), one precursor compound (FOSA), and four emerging PFEAs (NaDONA, GenX, 6:2 Cl-PFAES and 8:2 Cl-PFAES). The main physicochemical properties of these nine target compounds are provided in Table S1, in Supplementary Information. A stock solution containing a mixture of eight stable isotope standards (^13^C_4_-PFBA, ^13^C_2_-PFHxA, ^13^C_4_-PFOA, ^13^C_2_-PFDA, ^18^O_2_-PFHxS, ^13^C_4_-PFOS, ^13^C_3_-GenX and ^13^C_8_-FOSA) was used as a surrogate standard, while ^13^C_8_-PFOA and ^13^C_8_-PFOS were used as injection standards. All standards are high purity (*>*98 %) and were supplied from Wellington Laboratories (Guelph, ON, Canada). Polystyrene microplastics (PS-MPs, density of 1.05 g cm^−3^, size between 500 and 600 μm) selected in this study as model sorbent, were purchased from Polysciences Europe GmbH (www.polysciences.com). Further information on other chemicals, reagents, and materials used for the sorption experiments and analysis are detailed in Table S2.

### 2.2 In situ aging

**Fig. 1:**
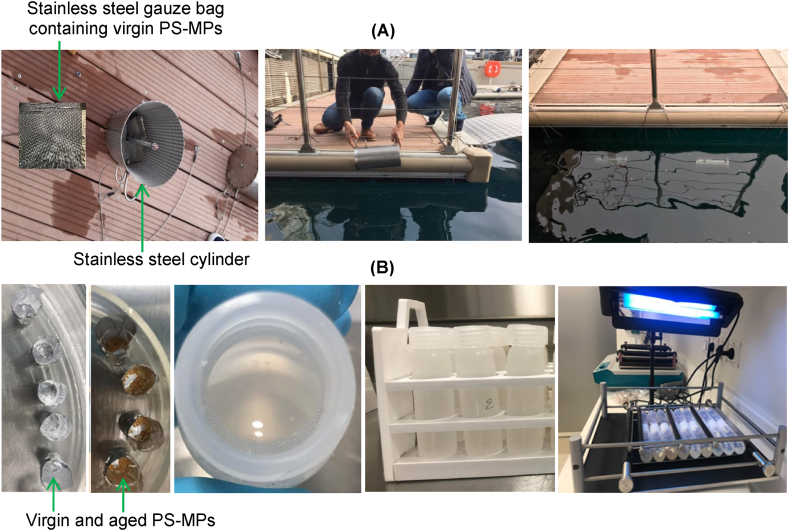
Experimental setup for the experiments examining the aging of PS-MPs in the field (A), and sorption of PFAS in the laboratory (B).

The aging of MPs was conducted with a protocol based on a previous study [48], which consists in submerging the virgin PS-MPs (500–600 μm) in seawater of the Monaco Harbour (NW Mediterranean, 43° 44′ 14.964″ N; 7° 25′ 44.364″ E) for 85 days, from December 2020 to February 2021. Details of the design of the aging experiment are provided in Text S1 and Fig. 1A.

### 2.3 Microplastic characterization

The surface functional groups and specific surface area (SSA) of PS-MPs samples before and after natural aging were analyzed by attenuated total reflectance-Fourier transform infrared spectrometer (ATR-FTIR; PerkinElmer, Spectrum 100, spectrometer Massachusetts, USA) and Brunauer-Emmett-Teller-N_2_ (BET-N_2_; Tristar 3000 porosimeter, Micromeritics Instrument Corporation, Norcross, GA, USA), respectively. Furthermore, the carbonyl index (CI), estimated by the absorbance of carbonyl relative to methylene peak (1730/1452) in FTIR spectra, was applied to describe the degree of surface oxidation of PS-MPs [49]. A scanning electron microscopy (SEM; JEOL 6300, JEOL Peabody, MA, USA) coupled with an energy dispersive X-ray spectroscopy (EDS) was used to examine surface morphology and elemental composition (weight %) of the virgin and aged PS-MPs, respectively. X-ray diffraction (XRD, D5000, Siemens) was also used to determine the crystallinity of virgin and aged PS-MPs.

### 2.4 Sorption experiment

The influence of natural aging on chemical sorption on MPs was investigated by comparing the sorption of PFAS on virgin and aged PS-MPs. Considering the background concentrations of PFAS and MPs in the environment, as well as the quantitation limits of the analytical method, 1 μg L^−1^ of each PFAS and 250 mg L^−1^ of MPs were selected for the sorption experiment. It has been reported that the concentrations of PFAS and MPs in the aquatic environment ranged from <3 × 10^−5^ to 51 μg L^−1^ and 5.51 ± 9.09 mg L^−1^, respectively [50,51]. The concentration of MPs applied in the present study was higher than the reported maximum concentrations in aquatic environment in order to handle the experiment more efficiently. As PFAS occur together in the environment, our experiments utilized sorbate mixtures (9 PFAS). The sorption experiment was performed for 7 days, correspond to the equilibrium sorption time for the studied PFAS as previously reported [37,38].

Specifically, the sorption experiment was designed as follows: six 50 mL polypropylene (PP) centrifuge tubes were filled with 40 mL of 1 μm-filtered seawater (FSW, ∼24 °C, pH ∼8, salinity ∼38–40 PSU, conductivity ∼55 mS cm^−1^) and spiked with 40 μL of a 1 mg L^−1^ standard mixture solution containing 9 PFAS to obtain an exposure concentration of 1 μg L^−1^ for each PFAS. The methanol volume (spiking solvent) was kept to less than 0.2 % of the total volume to prevent its effect on the sorption. Then, 10 mg of virgin or aged PS-MPs were added to the PP tubes to make its final concentration at 250 mg L^−1^. Each type of MP was tested in triplicate. Duplicate positive (with PFAS but PS-MPs-free) control tubes for each type of MP were run in parallel to assess the potential loss due to adsorption onto the tubes or volatilization. In addition, duplicate negative (with virgin PS-MPs but PFAS-free) control tubes were run to control possible release of PFAS from virgin PS-MPs. All tubes were shaken at 200 rpm for 7 days at room temperature (20–24 °C) (Fig. 1B). At the end of the experiment, after filtration of aqueous solutions through a 0.45 μm GF/F filter using a 50 mL glass syringe, 0.1 mL of each filtrate was transferred into a 250 μL PP vial along with 0.1 mL of 4 mM Ammonium acetate in methanol. To end, an aliquot of 10 μL of 200 pg μL^−1^ of ^13^C_8_-PFOA and ^13^C_8_-PFOS was added to the extract vial as injection standard before the analysis of residual PFAS.

### 2.5 Instrumental analysis

PFAS identification and quantification analyses were done with an Ultra-Performance Liquid Chromatography (UPLC) system coupled to a triple quadrupole mass spectrometer (Xevo TQD, Waters, Milford, MA, USA) operated in negative electrospray ionization (ESI) mode as described in our previous study [52]. Information about chromatographic conditions and MS parameters are detailed in Tables S3 and S4.

### 2.6 Quality assurance and quality control (QA/QC)

Following the quality assurance and quality control (QA/QC) procedures, caution was taken to evaluate any background contamination in experimental and instrumental blanks. Polypropylene tubes have been used instead of Teflon™ tubes to avoid any contamination and minimize PFAS sorption onto PP tubes walls. A positive control was carried out during the sorption experiment containing PFAS but PS-MPs-free. The loss of PFAS during the sorption was calculated and subtracted from the positive control. A negative control was also prepared by performing sorption experiment using virgin PS-MPs but PFAS-free showed that no detectable PFAS levels were released from the PS-MPs during the sorption process. Instrumental blank (methanol solvent injected at the same time as samples) was used and checked regularly to monitor carryover and background contamination. The instrumental blank contained no quantifiable analytes. In addition, to retain any PFAS contamination originating from the LC system that might interfere with the samples, an isolator column (ACQUITY XBridge C18 column (50 × 2.1 mm, particle size 3.5 μm, Waters, USA)) was inserted between the solvent mixer and injector. To ensure accuracy and precision, the quantification of the target PFAS was performed by isotope dilution mass spectrometry (IDMS) using the corresponding labelled surrogate standards. The calibration curve was set with 9 different concentrations ranging from 0.16 to 40 pg μL^−1^, R^2^ > 0.99. A midpoint of the calibration standard (10 pg μL^−1^) was injected at the beginning and end of each sample sequence to assure maintenance of sensitivity. Method detection limit (MDL) was calculated for each compound by determining the hypothetical peak area at 3 × signal-to-noise and quantifying it as if it was a real peak. It ranged from 10 ng L^−1^ (6:2 Cl-PFAES) to 64 ng L^−1^ (GenX).

### 2.7 Data analysis

The amount of PFAS sorbed on virgin or aged PS-MPs was estimated using the following equation (Eq. (1)):

(1) $$Q=\left(C_{\text{wc}}-C_{\text{wp}}\right)\times V/m$$

where Q (ng g^−1^) is the sorption amount of PFAS on virgin or aged PS-MPs, C_wc_ (ng L^−1^) is the concentration of PFAS in seawater positive control, C_wp_ (ng L^−1^) is the concentration of PFAS in seawater after sorption on virgin or aged PS-MPs, *V* (L) is the solution volume, and *m* (g) is the mass of PS-MPs used.

The partition coefficient (K_d_, L kg^−1^) between the water phase and the virgin or aged PS-MPs was calculated as follows (Eq. (2)):

(2) $$K_{d}=\left[\frac{Q\;}{C_{\text{wp}}\;}\right]\times 1000$$

Statistical analysis was conducted using SPSS software (Version 20.0), graph prism 5, and Microsoft Excel™ 2016. The sorption of PFAS onto virgin and aged PS-MS was carried out in triplicate (in duplicate for positive and negative controls) and the results are given as the mean values ± standard deviation (SD). Significant differences between group means were assessed with the Student (t) test at *p < 0.05* (one-tailed).

## 3 Results and discussion

Owing to the difficulties for investigating the sorptive properties of MPs in the field environment, most of the studies have been performed using virgin polymers or weathered polymers in laboratory experiments. However, the findings can be inherently different from those found in the environment. In this study, naturally aged MPs were employed instead of MPs artificially aged under laboratory conditions (i.e., aging using UV lamp, Fenton reaction, chrome acid oxidation, etc.) to consider the influence of natural aging conditions-including physical, chemical, and biological degradation on PFAS sorption onto PS-MPs.

### 3.1 Characterization of the PS-MPs

**Fig. 2:**
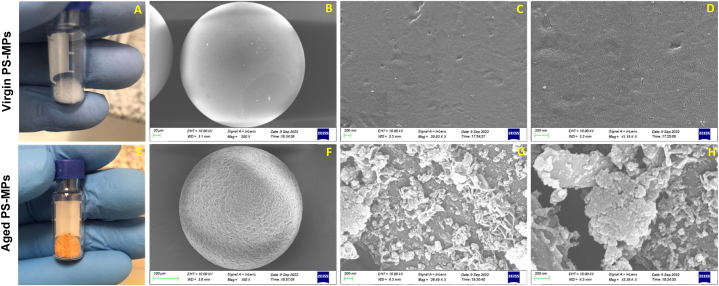
Optical pictures showing surface color of virgin and aged PS-MPs (A, and E), and SEM images (B, C, D, F, G, and H) showing the surface morphology of virgin (B, C, and D) and aged PS-MPs (F, G, and H) at different magnifications (200× (B), 20.03KX (C), 41.19KX (D), 150X (F), 26.89KX (G), and 43.38KX (H)).

**Fig. 3:**
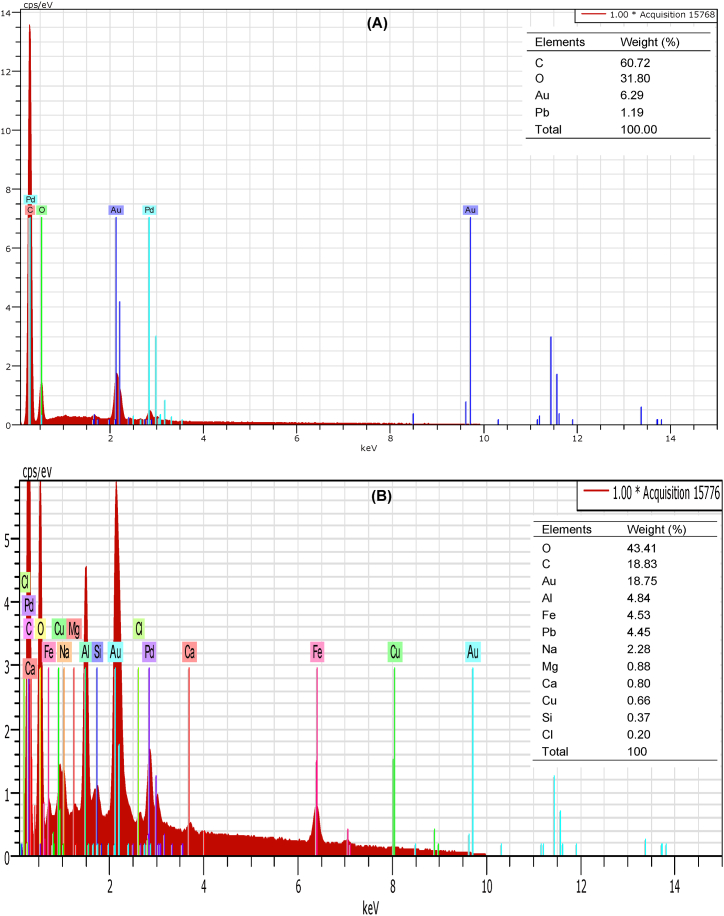
EDS spectrum of virgin (A) and aged PS-MPs (B).

Fig. 2 illustrates the optical and SEM images of the virgin and aged PS-MPs, which clearly shows that the surface color of aged PS-MPs turns orange. This is likely owing to the addition of functional groups containing oxygen on the surface of aged PS-MPs and/or the polyene structure that may have been produced during the aging process [53,54]. Analysis at different magnifications reveals that virgin PS-MPs appeared relatively smooth, flat, and uniform with the presence of small holes/pores and cracks. In contrast, the surfaces of aged PS-MPs are clearly rough and uneven with large fissures, pits and deposit of salt precipitates, which are general features of plastic degradation and biofilm formation [17,22,48,[55], [56], [57], [58], [59], [60]]. The presence of salt precipitates was confirmed when aged PS-MPs were examined by EDS (Fig. 3). The EDS spectrum shows a high percentage of oxygen (O), carbon (C) with small amounts of aluminum (Al), iron (Fe), sodium (Na), magnesium (Mg), calcium (Ca), silicon (Si), and chlorine (Cl). Former studies have also evidenced that biofilm on aged MPs was usually associated with this type of elements [22,61,62]. As shown in the optical pictures of aged PS-MPs (Fig. 2G and H), the non-homogeneous coating layer, with many rough structures stick on the surface might be attributed to patches of extracellular polymeric substances in biofilm as described in other studies [[63], [64], [65]].

Changes in the surface chemistry of the PS-MPs were monitored using the ATR-FTIR and EDS to analyze specific functional groups of the samples. As illustrated in Fig. S1, no evident spectral differences were noticed between virgin and aged PS-MPs. But, when we have calculated the CI, which is often used as a good indicator to quantify the degree of surface oxidation and aging [66], we found a 5-fold higher CI value for aged PS-MPs (0.92) than virgin PS-MPs (0.18). This results in an increase of hydrophilicity probably due to the photooxidation of PS-MPs under natural aging [67]. The CI estimated in this work fells in the range of CI reported in other different environmental MPs [68,69]. The additional formation of oxygen groups on aged PS-MPs spectra was also confirmed by EDS analysis (Fig. 3), with a relatively higher oxygen content on aged PS-MPs (43.4 %) compared to virgin PS-MPs (31.8 %). Other studies have also reported a rise in oxygen content on aged MPs [17,70,71]. By comparing the ATR-FTIR and EDS, we evidence a certain stage of oxidation owing to the formation of oxygen groups on aged PS-MPs. Although the PS-MPs contained in the stainless bag at 0.5 m depth were not exposed to much direct sunlight, the combination of low irradiation throughout the 85 days of exposure, reactive oxygen species and various organic and inorganic constituents can still drive some indirect photoaging. Also, as the PS-MPs were not chemically treated, the FTIR and SEM measurements can be affected by the presence of biofilm and the observed surface oxidation is likely related to the biomass. So, in our field experimental conditions, bio-oxidation seems to be the dominant factor affecting the surface oxidation of the PS-MPs in the field seawater.

Based on the SEM results above, we expected to obtain a higher SSA for aged PS-MPs than virgin PS-MPs, but the results of BET showed an unsignificant trend with slightly higher SSA for virgin PS-MPs (0.37 ± 0.15) than aged PS-MPs (0.29 ± 0.15). These results might be due to release of small size particles during the aging [72] and/or the deposition of sand, salt, and microbes that can occupy available sites on the surface of aged PS-MPs, as observed by the EDS results above. This is in good agreement with a previous study [73] reporting that BET surface area of polyamide MPs decreased after aging in river freshwater for 30 days, likely due to microbial biomass filling the pores. Analogous findings of decreasing SSA in aged MPs was also observed by several authors due to BET analysis limitations [13,16,[74], [75], [76]].

Aging may also modify the crystallinity of the polymer after a long residence time in marine and coastal environments [68,69]; thus, XRD was applied to compare the crystallization degree of PS-MPs before and after aging, and the result was presented in Fig. S2. As illustrate in Fig. S2, no evident spectral differences were noticed between virgin and aged PS-MPs, implying that natural aging during 85 days in seawater did not significantly affected the PS-MPs crystallinity. This agrees with the results of Junck et al. [16] who also reported no significant changes in the crystallinity between virgin and aged PE MPs. Corcoran et al. [77] reported that the degradation of plastics might happen quicker on terrestrial environments compared to the marine environment as lower exposure to UV radiation and lower mechanical erosion occurs in the ocean.

The surface charge of an adsorbent is a critical property that influences their capacity to sorb contaminants [78]. It is generally assessed by measuring the pH_PZC_, the pH at which the net charge of the adsorbent surface is neutral. Zhang et al. [79] revealed the pH_PZC_ of PS is 3.69. When the pH_PZC_ < pH of the medium then the surface of the MPs becomes negatively charged. Otherwise, its charge is positive. In this study, the sorption of PFAS onto virgin PS-MPs was done in seawater (pH ∼8), thus their surface was negatively charged. For aged PS-MPs the negative charge is expected to be higher since, based on EDS and ATR-FTIR, there are oxygen groups on the surface that are negative at this pH. A negative surface charge of the PS-MPs in water was also observed by Zhang et al. [79] at pH range of 3–11 and by Li et al. [80] for four sizes MPs. Generally, the surfaces of PS-MPs are negatively charged in marine (pH ∼8.1) or terrestrial (pH 6–7) waters [81,82]. The electrostatic repulsions between the negatively charged surfaces of PS-MPs and the anionic functional groups of PFAS (such as sulfonic acid or carboxyl groups) can be mitigated by the presence of bivalent cations like Ca^2+^ and Mn^2+^ in seawater. So, overall, indirect electrostatic interactions (e.g. through the presence of bivalent cations in seawater), along with hydrophobic interactions linked to C-F bonds, can play a significant role in the sorption behavior of anionic and neutral PFAS on PS-MPs.

### 3.2 Sorption of PFAS

**Fig. 4:**
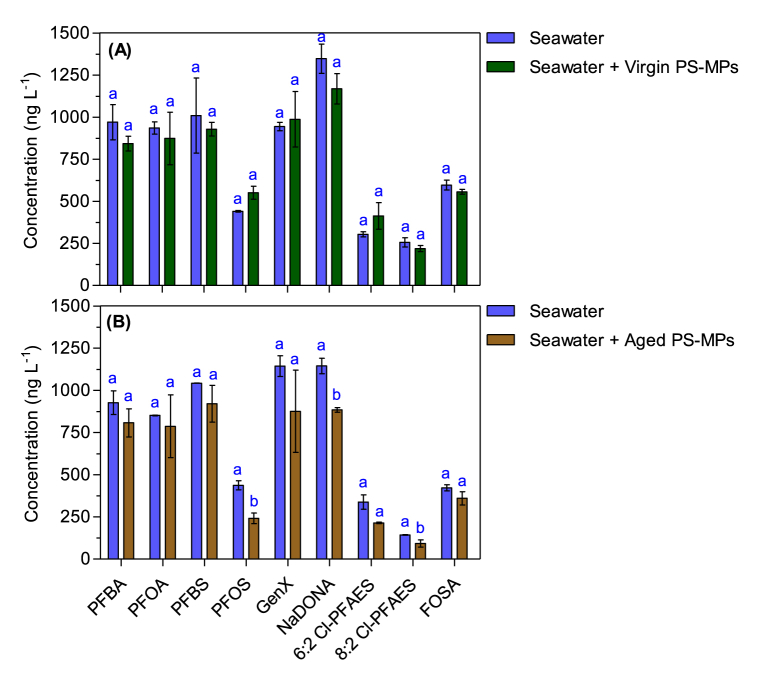
Average concentrations (ng L^−1^) of target analytes with and without the presence of virgin (A) or aged PS-MPs (B) in the exposure water (seawater) at 7 days. Lowercase letters on the top correspond to statistically different groups of data, calculated by the Student (t) test. Distinct lowercase letters indicate significant differences between groups (*p < 0.05*).

The actual concentrations (ng L^−1^) of the nine target analytes in the exposure water (seawater) at 7 days, with and without the presence of virgin or aged PS-MPs are shown in Fig. 4. Among the nine analyzed PFAS, the concentrations measured for 5 compounds (PFBA, PFOA, PFBS, GenX, and NaDONA) exceeded 800 ng L^−1^ in the positive control (seawater with PFAS but PS-MPs-free). This indicates that these PFAS were relatively constant during the entire exposure period. For the other four PFAS (PFOS, 6:2 Cl-PFAES, 8:2 Cl-PFAES, and FOSA) their concentrations were found lower compared to their initial nominal concentrations, ranging from 143 ng L^−1^ to 596 ng L^−1^. The loss of the latter long-chain PFAS in the water phase was likely due to their sorption *via* hydrophobic interactions onto the PP containers walls. This agrees with Zenobio et al. [83] who studied the sorption of PFAS on PP containers and indicated that hydrophobicity is the primary driving force influencing the sorption of long-chain PFAS on the PP containers. Although both PFOS and PFOA have the same number of carbons in their chain-length (Table S1), the loss of PFOS in the water phase was more important than PFOA (Fig. 4). This difference may be due to their different functional group, and that PFOS has an extra CF_2_ group compared to PFOA. Overall, these features imparts a higher hydrophobicity (and lower solubility) on PFOS, leading to the greater sorption onto PP containers. As mentioned in the QA/QC section (Text S4), any loss was taken into consideration during PFAS quantification. Regarding PFAS in seawater with virgin or aged PS-MPs (Fig. 4), the concentrations were generally lower than those in the positive control, except PFOS, GenX, and 6:2 Cl-PFAES which were higher in the seawater with presence of virgin PS-MPs than in positive control. These 3 PFAS were not sorbed onto the virgin PS-MPs.

#### 3.2.1 Sorption onto virgin PS-MPs

**Fig. 5:**
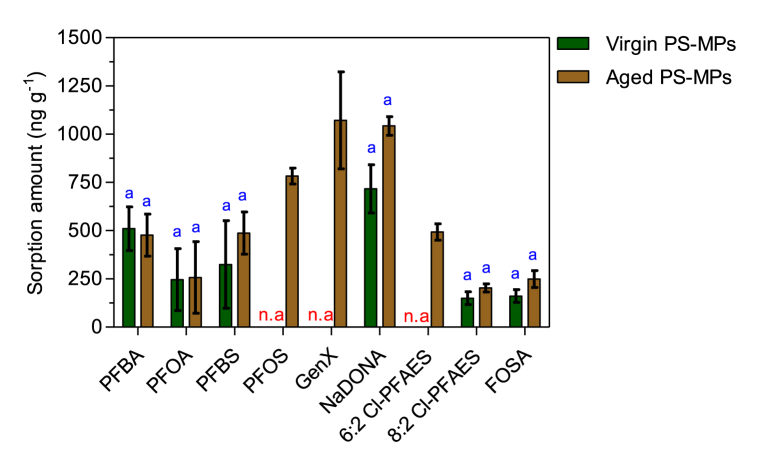
Sorption amount (ng g^−1^) of target analytes on the virgin and aged PS-MPs. Lowercase letters on the top correspond to statistically different groups of data, calculated by the Student (t) test. Distinct lowercase letters indicate significant differences between groups (*p < 0.05*). n.a. means no PFAS sorption was occurred in the related system.

The sorption amount of the nine PFAS on the virgin and aged PS-MPs are illustrated in Fig. 5. Six out of the 9 selected PFAS were sorbed on the virgin PS-MPs. The overall trend of sorption capacity of the six PFAS onto virgin PS-MPs was in the order: NaDONA (717 ng g^−1^) > PFBA (510 ng g^−1^) > PFBS (325 ng g^−1^) > PFOA (246 ng g^−1^) > FOSA (161 ng g^−1^) > 8:2 Cl-PFAES (150 ng g^−1^). The increasing trend of these PFAS (except NaDONA) was consistent with the decreasing trend of their molecular weight and molar volume values as illustrated in Fig. S3, which showed a significant negative correlation (R^2^ = 0.86–0.88, *p = 0.02*) between the sorption of the five PFAS and their molecular weight/molar volume. One review paper studying the sorption processes has already underlined that the molecular weight of hydrophilic contaminants is a key parameter in their sorption by MPs [84]. Hence, it seems that the higher affinity of the virgin PS-MPs toward short-chain PFAS could be related to their lower molecular weight or molar volume, which allows them to diffuse easily in the polymer. Regarding GenX (short-chain PFAS), we suggest that their relatively high hydrophilicity (Table S1) prevents their diffusion into the virgin PS-MPs. This agrees to some extent with the findings of Sun et al. [46] who also reported that GenX was not adsorbed onto the soil. Menéndez-Pedriza and Jaumot [84] reported that PS is a glassy polymer which presents a condensed structure but has high distance between polymeric chains which act as sorption sites. Furthermore, it has been reported that smaller compounds undergo reduced steric restrictions when diffusing into the polymer structure of MPs [25,27,82,85,86]. As an example, Dong et al. [82] found a quicker and high sorption of PFOA (compared to PFOS) onto natural clay because of reduced PFOA size compared to PFOS. Similar, Llorca et al. [25] attributed the unexpected high concentration of a short chain compound (PFPeA) sorbed into beached MPs to the steric effect. It is well known that the presence of micelles might also boost the partitioning of PFAS from water to solid surfaces, in particularly for the short-chain PFAS with a higher critical micelle concentration (CMCs) than long-chain PFAS (Table S1) [27,87]. However, here it seems unlikely that micelles formation might had a significant role in the adsorption because the tested concentrations (1 μg L^−1^ for each PFAS) are far below their CMC values.

Except FOSA, the PFAS studied here with a pKa <2 (Table S1) indicates that are present in anionic forms at the pH (∼8) of the seawater. Hence, we expect an electrostatic repulsion among the negatively charged PS-MPs and the anionic PFAS, and a higher sorption (mainly by hydrophobic interaction) for FOSA as with an estimated pKa of 6.24 [88], it coexists in the anionic and neutral form. We also speculate that the low sorption amount of FOSA on PS-MPs observed here may be due to the steric limitation caused by the bond rotation of the benzene rings of the PS-MPs significantly reducing the free volume among chains and thus, limiting the FOSA diffusion into MPs pores [37]. The reason why PFOS and 6:2 Cl-PFAES are not adsorbed onto the virgin PS-MPs is not yet known. We consider that the competition between the nine PFAS mixture for the binding sites on the PS-MPs surfaces might be the reason, but further studies should be performed. Wang et al. [37] attributed the absence of PFOS sorption on the virgin PS-MPs to the electrostatic repulsion among the negative surface of PS particles and the anionic form of PFOS.

Generally, the mechanisms by which PFAS sorb on virgin PS-MPs is still largely unknown. Based on the discussion above, we speculate that the steric hindrance mechanism alone is insufficient to explain the sorption behavior of PFAS on the virgin PS-MPs, particularly for the long-chain (and high MW) PFAS, e.g., like 8:2 Cl-PFAES. In addition, FOSA with an estimated pKa of 6.24 partially exists also in neutral form in the seawater, and this may determine the principal role of hydrophobic interactions among PS-MPs and FOSA. Therefore, we presume that hydrophobic interactions also influence the sorption of long-chain PFAS on the virgin PS-MPs. The accessible information on PFAS sorption on virgin PS-MPs showed that hydrophobic interaction is among the dominant mechanisms that governed the sorption of PFAS on PS-MPs [14]. In addition, since PS is a glassy polymer, other interaction types including electrostatic interaction, Van-der-Waals, ionic, and covalent bonds may occurs [76,[89], [90], [91]].

#### 3.2.2 Sorption onto aged PS-MPs

As illustrated in Fig. 5, most PFAS showed enhanced sorption (although not significant) on aged PS-MPs compared to the virgin PS-MPs. For instance, 8:2 Cl-PFAES, NaDONA/PFBS, and FOSA exhibited a 1.4-, 1.5-, and 1.6-fold augmentation in sorption on aged PS-MPs. In addition, PFOS, GenX, and 6:2 Cl-PFAES which were not sorbed onto the virgin PS-MPs have undergone different sorption capacities on the aged PS-MPs, which ranged from 493 ng g^−1^ to 1072 ng g^−1^. The maximum sorption during the 7-d exposure was observed for PFOA alternatives, namely GenX (1072 ng g^−1^) and NaDONA (1043 ng g^−1^). Our results are in good agreement with many previous studies that have also demonstrated that aging process, either naturally or artificially can increase the sorption of a broad type of organic and inorganic contaminants, including PFAS on different MPs [21,39,59,92]. For instance, a study by Bhagwat et al. [17] showed that naturally aged microfibers of polypropylene, polyethylene, nylon and polyester sorbed more PFOS than the virgin ones. Also, Ateia et al. [39] found an enhancement in the uptake of PFOS, PFOA and GenX by MPs pre-exposed with natural organic matter from lake water.

It is difficult to establish the main causes that enhanced the association of PFAS on aged PS-MPs. We speculate that this can be attributed to the morphological, chemical, and biological changes that PS-MPs undergo during aging in seawater for 85 days as shown by SEM and EDS analysis. The morphological changes (i.e., rough surface, cavities, pits, Fig. 2) on aged PS-MPs indeed create more sorption sites, enhancing the diffusion and sorption of PFAS. Additionally, the introduction of oxygen groups can modify the functional groups on their surface increasing their ability to sorb PFAS through electrostatic and hydrogen-bonding interactions [93]. This is consistent with previous studies [13,21,90,94,95] reporting that the morphological and chemical changes of MPs are important parameters in the sorption of pollutants. Biofilm growth on MPs can also facilitate the sorption of PFAS [15]. As carboxyl and sulfonic acid groups of PFAS are negatively charged in seawater, PFAS are repulsed on the negative charged PS-MPs. Nevertheless, biofilms can act as a “mediator” to lighten these electrostatic interactions through the EPS (main constituent of biofilms), compiled with a mix of exopolysaccharides, proteins, nucleic acids and lipids [15,92]. Yan et al. [96] described that the rough surface of EPS and their richness with proteins and functional groups may provide sorption sites for PFAS. In addition, the strong hydrophobicity of EPS can enhance the adsorption of the C–F bonds of PFAS [96]. On the other hand, the high sorption capacity of short-chain PFAS (GenX and NaDONA) on aged PS-MPs observed in the present study (Fig. 5) may also suggest their diffusion through the biofilm. This agrees with Li et al. [97] who reported that the paths for diffusion of water and nutrients in the biofilm may facilitate the transfer of short-chain PFAS. Overall, natural aging modifies the sorption properties of PS-MPs for PFAS *via* the altered surface properties (e.g. rough surface, introducing oxygen-containing functional groups, etc.) and the presence of biofilms.

**Fig. 6:**
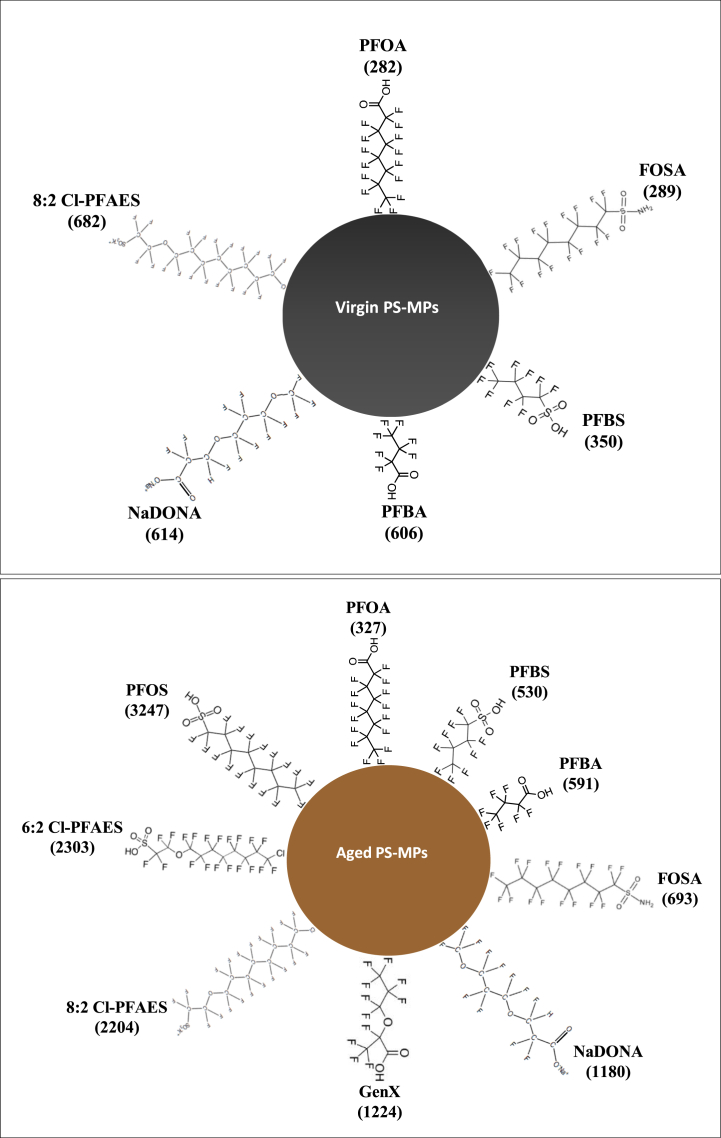
The partition coefficient (K_d_, L kg^−1^) between the seawater phase and the virgin or aged PS-MPs. The compounds are ordered in clockwise direction with increasing sorption. No sorption had occurred for PFOS, GenX and 6:2 Cl-PFAES on the virgin PS-MPs.

The sorption affinity of PS-MPs for PFAS can also be expressed using the solid-water partition coefficient (K_d_), and the octanol water coefficient (K_ow_), which are parameters frequently used as a measure of the hydrophobicity of compounds [98]. The K_d_ of the selected PFAS sorbed on the virgin and aged MPs are displayed in Fig. 6. The range of the K_d_ values for virgin and aged PS-MPs was 282–682 L kg^−1^ and 327–3247 L kg^−1^, respectively. Under both conditions, PFOA had the smallest K_d_. The order of K_d_ values for virgin (8:2 Cl-PFAES > NaDONA > PFBA > PFBS > FOSA > PFOA) and aged (PFOS >6:2 Cl-PFAES >8:2 Cl-PFAES > GenX > NaDONA > FOSA > PFBA > PFBS > PFOA) PS-MPs was not consistent with their K_ow_ (lack of correlations, Fig. S4). This implies that hydrophobicity is not the major factor controlling the amount of PFAS sorbed on PS-MPs and that molecular size is a better descriptor for the sorption process of PFAS than the K_ow_, as mentioned above. Best on our information, there is no current sources available reporting K_d_ values for PFAS sorption onto aged PS-MPs. Llorca et al. [38] reported K_d_ values in seawater for PFAS (PFBA, PFOA, PFBS, PFOS, and FOSA) adsorption onto virgin PS-MPs (10 μm) which ranged from not determined to 619 L kg^−1^. The K_d_ values of the current study falls within this range, but lower than K_d_ values for other non-polar organic compounds (e.g. polycyclic aromatic hydrocarbons, chlorobenzene, nitrobenzene) sorbed on different types of MPs [76,81,98,99].

## 4 Conclusions

In this study, the sorption behavior of PFAS on virgin and aged PS-MPs was examined to explore how natural aging affects the sorption behavior of the PS-MPs for PFAS. The physical and chemical surface properties of PS-MPs were affected after aging resulting in an enhancement of PFAS sorption on aged PS-MPs compared to the virgin PS-MPs. The interactions between PS-MPs and emerging contaminants in seawater are indeed complex. While laboratory studies provide valuable insights, real-world conditions introduce many additional variables that can affect these interactions, such as salinity, temperature, ionic strength, oxygen content, dissolved organic matter, pollutant competition, complex formation, among other. Therefore, more research (e.g. multi-solute sorption experiments) should focus on the effect of these factors on the partitioning of PFAS onto PS-MP. Moreover, results from this study indicates that biofilms play a crucial role in enhancing the sorption of PFAS on aged PS-MPs. Thus, future research should focus on characterizing biofilm community structures (e.g., using metagenomics and microbiome analysis) on aged PS-MP and linking these findings to the PFAS sorption behaviors. This is a crucial step to understand the behavior, fate, and toxicity of these two co-existing emerging pollutants.

## CRediT authorship contribution statement

**Badreddine Barhoumi:** Conceptualization, Investigation, Methodology, Data curation, Writing – original draft, and Writing – review & editing. **Marc Metian:** Conceptualization, Writing – review & editing, Resources. **Carlos M. Alonso-Hernández:** Conceptualization, Writing – review & editing, Resources. **Hrissi K. Karapanagioti:** Conceptualization, Writing – review & editing, Methodology, Investigation, Resources. **François Oberhaensli:** Designed experiments, Writing – review & editing, Methodology. **Nikolaos Mourgkogiannis:** Writing – review & editing, Data curation. **Philippe Bersuder:** Writing – review & editing, Investigation, Resources. **Imma Tolosa:** Supervision, Conceptualization, Methodology, Writing – review & editing, Investigation, Validation.

## Data availability

All data generated or analyzed during this study are included in this published article and its supplementary information files.

## Funding

This research work has been funded by the US through the Peaceful Uses Initiatives (PUI) program under the project of “Implementation of a comprehensive sampling and analytical methodology to determine and trace oil pollution in marine waters (Phase II – Marine plastics: tackling the challenge using nuclear applications)”.

## Declaration of Competing Interest

The authors declare that they have no known competing financial interests or personal relationships that could have appeared to influence the work reported in this paper.
