# Supplementary material for: Insight into the effect of natural aging of polystyrene microplastics on the sorption of legacy and emerging per- and polyfluorinated alkyl substances in seawater
Source: Heliyon. 2024 Nov 16;10(23):e40490. doi: 10.1016/j.heliyon.2024.e40490 (PMC11626057; doi:10.1016/j.heliyon.2024.e40490)
Supplement: Multimedia component 1 [file mmc1.docx]

*SUPPLEMENTARY INFORMATION*

**Insight into the effect of natural aging of polystyrene microplastics on the sorption of legacy and emerging per- and polyfluorinated alkyl substances in seawater**

Badreddine Barhoumi ^a,^ *, Marc Metian ^a^, Carlos M. Alonso-Hernández ^a^, François Oberhaensli ^a^, Nikolaos Mourgkogiannis ^b^, Hrissi K. Karapanagioti ^b^, Philippe Bersuder ^a^, Imma Tolosa ^a,^ *

*^a^ IAEA Marine Environment Laboratories, 4a Quai Antoine 1er, 98000 Monaco, Principality of Monaco*

*^b^ Department of Chemistry, University of Patras, 26504 Patras, Greece*

*Corresponding authors

E-mail: [barhoumibadredine@yahoo.fr](mailto:barhoumibadredine@yahoo.fr)

E-mail: I.Tolosa@iaea.org

**The supplementary information contains 11 pages, and includes one text, four figures and four tables.**

**Table S1.** Major physicochemical properties of investigated PFAS.

| Compounds name | Structure | Chemical  formula | Carbon chain length | MW (g/mol) | Solubility  (mol/L) ^a^ | Log K_ow_ ^a^ | pKa | Molar  volume (cm^3^) ^a^ | CMC (mol/L) ^c^ |
| --- | --- | --- | --- | --- | --- | --- | --- | --- | --- |
| Perfluorobutanoic acid (PFBA) |  | C_3_F_7_CO_2_H | 4 | 214.0 | 2.83 x 10^-3^ | 2.53 | -0.21 ^a^ | 127 | 0.71 to 0.80 |
| Perfluorooctanoic acid (PFOA) | 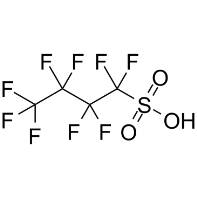 | C_7_F_15_CO_2_H | 8 | 414.1 | 5.52 x 10^-4^ | 5.94 | 0.34 ^a^ | 237 | 0.008, 0.01 |
| Perfluorobutane sulfonic acid (PFBS) |  | C_4_F_9_SO_3_H | 4 | 300.1 | 5.02 x 10^-3^ | 2.77 | -1.61 ^a^ | 162 | 0.022 |
| Perfluoroooctane sulfonic acid (PFOS) | 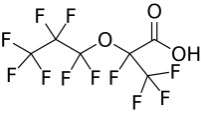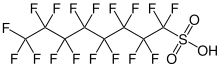 | C_8_F_17_SO_3_H | 8 | 500.1 | 4.09 x 10^-4^ | 5.95 | -1.64 ^a^ | 272 | 0.0002 |
| Hexafluoropropylene oxide dimer acid (GenX) |  | C_6_HF_11_O_3_ | 6 | 330.1 | 1.41 x 10^-2^ | 5.1 | -0.73 ^a^ | 189 | 0.175 |
| Sodium dodecafluoro-3H-4,8-dioxanonanoate (NaDONA) | **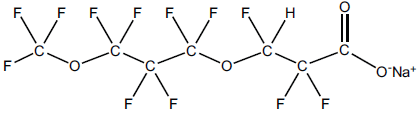**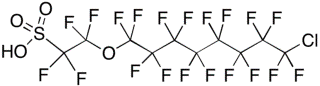 | C_7_HF_12_O_4_Na | 7 | 400.1 | 1.82 x 10^-3^ | 5.78 | -0.22 ^a^ | 217 | - |
| 6:2 chlorinated polyfluoroalkyl ether sulfonate (6:2 Cl-PFAES) |  | C_8_HF_16_ClSO_4_ | 8 | 532.6 | 4.41 x 10^-4^ | 5.71 | 1.58 ^a^ | 284 | - |
| 8:2 chlorinated polyfluoroalkyl ether sulfonate (8:2 Cl-PFAES) | **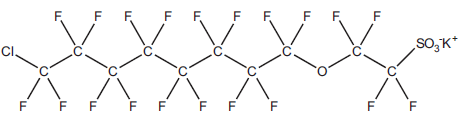**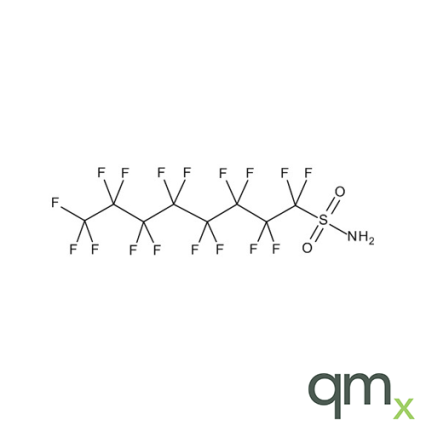 | C₁₀HF_20_ClSO₄ | 10 | 632.6 | 2.16 x 10^-5^ | 7.41 | 1.96 ^a^ | 339 | - |
| Perfluorooctane sulfonamide (FOSA) |  | C_8_H_2_F_17_NO_2_S | 8 | 499.1 | 3.88 x 10^-7^ | 6.55 | 6.24 ^b^ | 278 | - |
| ^a^US EPA ([*https://comptox.epa.gov/dashboard/chemical_lists/pfasmaster*](https://comptox.epa.gov/dashboard/chemical_lists/pfasmaster)); ^b^Rayne and Forest [1]; ^c^Salawu et al. [2]; MW = Molecular weight; Log K_ow_ = octanol water coefficient; pKa = acid-dissociation constant; CMC = Critical micelle concentration. | | | | | | | | | |

**Table S2.** List of chemicals, reagents and materials used in this study.

| **Chemicals, reagents, and materials** | **Specification by supplier** | **Supplier** | **Purity** |
| --- | --- | --- | --- |
| Methanol | for LC-MS (optima grade) | Fisher Scientific, France | > 99.9% |
| Ammonium acetate | for LC-MS | Fisher Scientific, France | > 98% |
| Ultrapure water (18.2 MΩ⋅cm) | supplied by Milli-Q IQ 7000 | Merck, France | - |
| Micropipette | eppendorf 10-200 µL | Fisher Scientific, France | - |
| Micropipette | eppendorf 100-1000 µL | Fisher Scientific, France | - |
| 1 mL polypropylene vials | with screw cap in polypropylene | Agilent Technologies, France | - |
| 250 µL polypropylene vials | with screw cap in polypropylene | Agilent Technologies, France | - |
| 50 mL polypropylene centrifuge tubes | with screw cap in polypropylene | Fisher Scientific, France | - |
| Glass fiber filters (GF/F) | Whatman™, 0.7 µm, 47-mm diameter | VWR, France | - |
| 50 mL luer lock glass syringe | FORTUNA^®^ OPTIMA^®^ | VWR, France | - |
| Analytical balance | Sartorius LP2200S | Sartorius, France | - |
| Vortex Mixer | Analog Vortex Mixer | VWR, France | - |
| pH meter | 913 pH Meter | Metrohm, Switzerland | - |
| Light meter | LI-COR Li-250A | Biosciences-Biotechnology, USA | - |
| Thermometer | Hanna HI98509 | MERCK, France | - |
| Conductometer | 912 Conductometer | Metrohm, Switzerland | - |
| Orbital shaker | PSU-20i | VWR, France | - |
| Stainless steel cylinders | Height=30 cm, OD=15 cm, with closed top and bottom  Inner mesh: Opening=0.3 mm, wire=0.20 mm  Outer support: Perforated sheet, hole=5.0 mm, thickness=1.0 mm | Silduksfabriken, Sweden | - |
| Stainless Steel gauze | Alfa Aesar™, 100 mesh woven from 0.11 mm (0.0045 in.) dia. wire, Type 316, 300 x 300 mm | Fisher Scientific, France | - |

**Text S1. In situ aging**

Sufficient amounts of PS-MPs were placed in a stainless-steel gauze bag of 100 µm mesh and introduced in a stainless-steel cylinder of 300 µm mesh to facilitate removal of fouling organisms and maintain the flow of water to the PS-MPs whilst avoiding their loss. The cylinder was then fixed on ropes and deployed 0.5 m below the marine surface water. At the end of the exposure period, the bag was collected from the cylinder, rinsed with seawater to remove debris, wrapped in aluminium foil and transported to the laboratory. There, the aged PS-MPs were rinsed with ultrapure water to remove the unattached materials and dried at room temperature under the fume hood before the sorption experiments and characterization. Environmental metadata such as light intensity, temperature, pH, salinity, and conductivity were periodically measured on-site at 0.5 m during the exposure period, which ranged from ~16 to ~70 µmol s^-1^ m^-2^, 13.7 to 15.8 °C, 7.9 to 8.1, 36.2 to 36.9 PSU, and 54.7 to 56.2 mS cm^-1^, respectively. Because the mesh of the stainless-steel gauze bag used to store the PS-MPs was 100 µm and that of the stainless-steel cylinder 0.3 mm., only a small percentage of light should reach the PS-MPs at a depth of 0.5 meters. The amount of light penetrating to the stainless-steel cylinder and stainless-steel gauze was estimated through measurements of the light outside of the stainless-steel cylinder (outside of the water) and inside the combined stainless steel cylinder and gauze. As a result, only 10% of the light irradiated outside of the cylinder reached the MPs inside the gauze and cylinder. Based on that, PS-MPs were exposed to a light intensity of ~1.6 to 7 µmol s^-1^ m^-2^.

**Table S3.** UPLC-MS/MS instrument conditions for analysis of the target analytes.

| **Parameters** | | **Conditions** |  |  |  |  |  |  |  |
| --- | --- | --- | --- | --- | --- | --- | --- | --- | --- |
| *Liquid chromatography (LC)* | |  |  |  |  |  |  |  |  |
| Instrument | | UPLC Acquity, Waters |  |  |  |  |  |  |  |
| Analytical column | | Waters BEH C18 column (50 x 2.1 mm, particle size 1.7 µm) | | | | | | | |
| Delay column | | Waters XBridge C18 column (50 x 2.1 mm, particle size 3.5 µm) | | | | | | | |
| Mobile phase | | A) 10 mM ammonium acetate in Milli-Q water, (B) 10 mM ammonium acetate in methanol | | | | | | | |
|  | Gradient | Time (min) | 0 | 0.5 | 3 | 14 | 15 | 15.1 | 17 |
|  |  | B (%) | 10 | 10 | 50 | 100 | 100 | 10 | 10 |
|  | Column temperature | 50 °C |  |  |  |  |  |  |  |
|  | Flow rate | 0.3 mL/min |  |  |  |  |  |  |  |
|  | Injection volume | 10 μL |  |  |  |  |  |  |  |
| *Tandem mass spectrometry (MS/MS)* | |  |  |  |  |  |  |  |  |
| Instrument | | Triple quadrupole mass spectrometer (Xevo TQD, Waters, USA) | | | | | | | |
| Ionization mode | | Electrospray ionization (ESI) in negative mode | | | | | | | |
|  | Scan type | Multiple Reaction Monitoring (MRM) | | | | | | | |
|  | Desolvation Temperature | 350 °C |  |  |  |  |  |  |  |
|  | Source temperature | 150 °C |  |  |  |  |  |  |  |
|  | Source Gas | Nitrogen |  |  |  |  |  |  |  |
|  | Desolvation gas flow | 1000 L/h |  |  |  |  |  |  |  |
|  | Cone gas flow | 20 L/h |  |  |  |  |  |  |  |
|  | Ion spray voltage | -1000 V |  |  |  |  |  |  |  |
|  | Data system | TargetLynx Software |  |  |  |  |  |  |  |

**Table S4.** Ion transitions and surrogate standards used for target analytes quantification by UPLC-MS/MS.

| **Retention time (min)** | **Compound Name** | **Parent Ion (m/z)** | **Daughter Ion (m/z)** | **Cone voltage (V)** | **Collision energy (eV)** | **Surrogate standard** |
| --- | --- | --- | --- | --- | --- | --- |
| 0.1 - 6.15 | PFBA | 213 | 169* | 10 | 9 | ^13^C_4_-PFBA |
|  | PFBS | 298.9 | 79.8* | 60 | 32 | ^18^O_2_-PFHxS |
|  |  | 298.9 | 98.8 | 60 | 30 |  |
|  | GenX | 285 | 169* | 12 | 8 | ^13^C_3_-GenX |
|  |  | 285 | 185 | 12 | 20 |  |
| 6.15 – 9.2 | NaDONA | 377 | 251* | 10 | 10 | ^13^C_2_-PFHxA |
|  |  | 377 | 84.8 | 23 | 32 |  |
|  | PFOA | 413 | 368.9* | 22 | 10 | ^13^C_4_-PFOA |
|  |  | 413 | 168.9 | 22 | 20 |  |
|  | PFOS | 499 | 98.9* | 74 | 46 | ^13^C_4_-PFOS |
|  |  | 499 | 80 | 80 | 48 |  |
|  | 6:2 Cl-PFAES | 531 | 351* | 40 | 25 | ^13^C_4_-PFOS |
|  |  | 531 | 83 | 60 | 25 |  |
| 9.2 – 17 | FOSA | 498 | 78* | 56 | 34 | ^13^C_8_-FOSA |
|  |  | 498 | 169 | 56 | 34 |  |
|  | 8:2 Cl-PFAES | 631 | 451* | 23 | 30 | ^13^C_2_-PFDA |
|  |  | 631 | 83 | 60 | 30 |  |
|  | ^13^C_4_-PFBA | 217 | 172* | 10 | 6 | - |
|  | ^18^O_2_-PFHxS | 403 | 84* | 45 | 35 | - |
|  |  | 403 | 103 | 45 | 33 | - |
|  | ^13^C_3_-GenX | 287 | 169* | 12 | 8 | - |
|  | ^13^C_2_-PFHxA | 315 | 270* | 26 | 10 | - |
|  |  | 315 | 119 | 10 | 9 | - |
|  | ^13^C_4_-PFOA | 417 | 372* | 6 | 10 | - |
|  |  | 417 | 172 | 10 | 9 | - |
|  | ^13^C_4_-PFOS | 503 | 80* | 60 | 54 | - |
|  |  | 503 | 99 | 60 | 53 | - |
|  | ^13^C_8_-FOSA | 498 | 78* | 56 | 34 | - |
|  |  | 498 | 169 | 56 | 34 | - |
|  | ^13^C_2_-PFDA | 515 | 470* | 6 | 10 | - |
|  | ^13^C_8_-PFOA | 420.9 | 375.8* | 22 | 8 | - |
|  |  | 420.9 | 172 | 10 | 18 | - |
|  | ^13^C_8_-PFOS | 507 | 80* | 65 | 42 | - |
|  |  | 507 | 99 | 65 | 76 | - |
| *Asterisks mark the product ions which were used as quantifier, whereas the other product ions were used as qualifier. | | | | | | |

Virgin PS-MPs

Aged PS-MPs


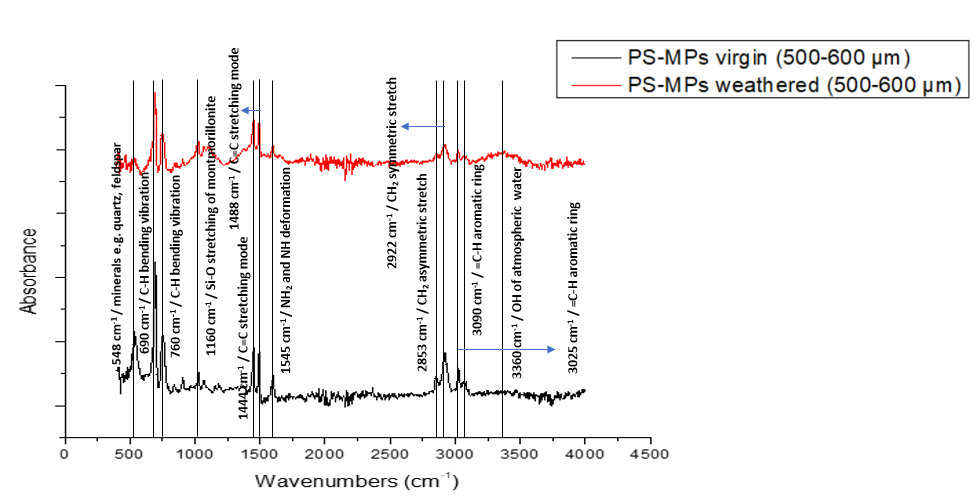


**Figure S1.** ATR-FTIR spectrum of virgin and aged PS-MPs.

Virgin PS-MPs

Aged PS-MPs


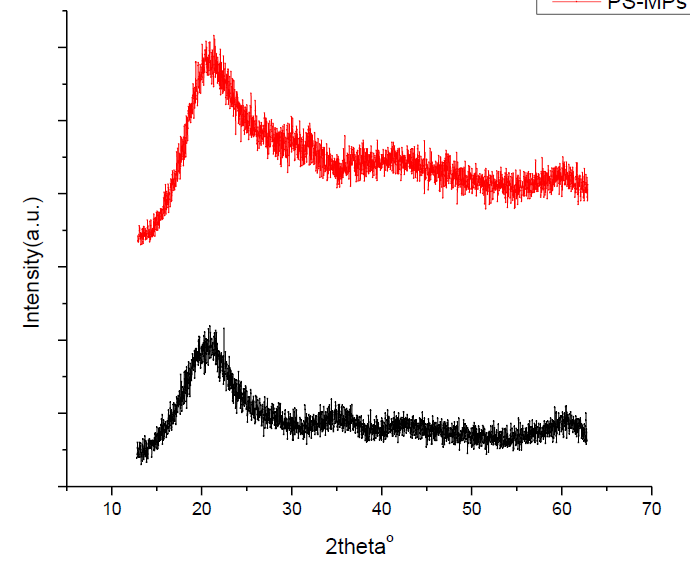


**Figure S2.** XRD spectrum of virgin and aged PS-MPs.

**Figure S3.** Relationships between the molecular weight (MW) (A) and molar volume (B) and concentrations of PFBA, PFOA, PFBS, 8:2 Cl-PFAES and FOSA sorbed on the virgin PS-MPs.

**Figure S4.** The correlations between Log K_d_ and Log K_ow_ values for the nine PFAS sorbed on the virgin (A) and aged (B) PS-MPs.

**References**

[1] S. Rayne, K. Forest, A new class of perfluorinated acid contaminants: Primary and secondary substituted perfluoroalkyl sulfonamides are acidic at environmentally and toxicologically relevant pH values, J. Environ. Sci. Health, Part A: Toxic/Hazard. Subst. Environ. Eng. 44 (2009) 1388–1399.

[2] O.A. Salawu, C.I. Olivares, A.S. Adeleye, Adsorption of PFAS onto secondary microplastics: A mechanistic study, J. Hazard. Mater. 470 (2024) 134185.
